# Muscle training‐induced bilateral brachial plexopathy in an adolescent with sporadic hereditary neuropathy with liability to pressure palsies

**DOI:** 10.1002/brb3.783

**Published:** 2017-07-27

**Authors:** Minori Kodaira, Satoshi Kodama, Yui Kamijo, Tomoki Kaneko, Yoshiki Sekijima

**Affiliations:** ^1^ Department of Medicine (Neurology and Rheumatology) Shinshu University School of Medicine Matsumoto Japan; ^2^ Department of Neurology Saku Central Hospital Advanced Care Center Saku Japan; ^3^ Department of Radiology Shinshu University School of Medicine Matsumoto Japan

**Keywords:** adolescent, brachial plexopathy, hereditary neuropathy with liability to pressure palsies, muscle training, sporadic

## Abstract

There have been few studies regarding physical training‐induced peripheral nerve dysfunction in patients with hereditary neuropathy with liability to pressure palsies (HNPP), with the exception of soldiers that trained intensively. Here, we report a 15‐year‐old boy without family history of HNPP who developed bilateral painless brachial plexopathy following short‐term barbell and plank training during a school baseball club activity. Muscle training‐induced painless brachial plexopathy could be an initial symptom and may be underdiagnosed in adolescents with sporadic HNPP.

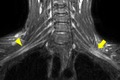

Exercise is known to have various benefits in healthy subjects (Garber et al., 2011) and is becoming more popular in individuals of all ages. Exercise may also lead to gains in muscle strength and physical function in patients with Charcot–Marie–Tooth disease (CMT), which is the most common hereditary neuropathy. However, a limited amount of evidence on the optimal exercise modality and intensity has been accumulated to date (Sman et al., 2015). Hereditary neuropathy with liability to pressure palsies (HNPP), which is a *peripheral myelin protein 22* (*PMP22*) gene‐related genetic disorder similar to CMT type 1A, is characterized by repetitive painless mononeuropathies and plexopathies triggered by minor trauma of the peripheral nerves (Mouton et al., 1999). However, there have been few studies regarding physical training‐ and/or exercise‐induced peripheral nerve dysfunction in patients with HNPP, with the exception of soldiers who have trained and/or exercised intensively (Horowitz, Spollen, & Yu, 2004; Kim, 2014; Mäkelä, Ramstad, Mattila, & Pihlajamäki, 2006). Here, we report the case of an adolescent with sporadic HNPP who developed bilateral painless brachial plexopathy following short‐term barbell training and plank exercise.

A 15‐year‐old boy, was the second child of healthy nonconsanguineous parents; he had a healthy sibling. He was born at term after a normal pregnancy and showed normal development. At 15 years of age, he began muscle training as part of a school baseball club activity. He developed weakness and numbness in the left upper extremity without pain during the new training regime, which involved keeping his knees bent with a 40‐kg barbell on his shoulder 10 times for 1 min each. Having repeated the same training the following day, his symptoms exacerbated. He visited two orthopedic clinics and the orthopedics department of a hospital, but no diagnosis was made. Two weeks later, he began performing plank exercise, an isometric core strength exercise that involved supporting the body on the forearms and toes for 1 min three times a day, which was extended to 3 min three times a day after a week. During the plank exercise, proximal upper extremity weakness developed on the right side without pain. The patient was admitted to the neurology department of another hospital 1 month after the onset of his first neurological symptoms. On admission, he showed diffuse left side and proximal right side upper extremity weakness (i.e., supraspinatus, 4/4; infraspinatus, 4/4; deltoid, 5/4; biceps, 4/4; triceps, 4/4; wrist extensor, 5/4; wrist flexor, 5/4; finger extensor, 5/4; abductor pollicis brevis, 5/4; and abductor digit minimi, 5/4 on the Medical Research Council Scale [MRC, 0–5]). The patient also had unapparent leg‐muscle weakness. Mild sensory disturbance of superficial sensation was detected in the left forearm. The tendon reflex in the upper extremities was diffusely reduced, but that in the legs was preserved. Long tract signs were unapparent. The results of blood and cerebrospinal fluid (CSF) analyses, including serum antiganglioside antibodies and CSF protein, were unremarkable. Cervical magnetic resonance (MR) imaging did not detect any spinal cord lesions; however, maximum intensity projection images on coronal T2‐weighted imaging (STIR; short TI inversion recovery) showed enlargement and hyperintensity of the bilateral brachial plexuses, especially on the left side (Figure 1). A nerve conduction study (NCS) indicated multifocal conduction slowing with neither conduction block nor temporal dispersion in both upper and lower extremities (Table 1). Electromyography revealed a mild reduced recruitment pattern without active or chronic denervation potentials in the affected muscles (i.e., left biceps, triceps, and extensor carpi radialis longus muscles). The patient was suspected with immune‐mediated neuropathy involving bilateral brachial plexuses and was treated with intravenous steroid therapy, which did not improve his neurological symptoms. Two months after symptom onset, he was referred to our hospital. Neurologically, MRC grade 4 muscle weakness was detected in the bilateral supraspinatus and infraspinatus muscles; however, strength in other muscles improved. Mild sensory disturbance remained in the left C6 dermatome. Follow‐up NCS showed increase in F‐wave occurrence in the upper extremities (Table 1), which suggested improved conduction block at the brachial plexuses. Exacerbation of parts of F‐wave latencies in the upper extremities between the 1st and 2nd studies might be due to fluctuation in F‐wave latencies (Pinheiro, Manzano, & Nóbrega, 2008) and/or the examination interval of 1 month, which was long enough to resolve conduction block, but too short to improve conduction slowing satisfactorily (Fowler, Danta, & Gilliatt, 1972). Stimulation at the Erb's point was not performed because weakness of the muscles innerved by the median and ulnar nerves was already ameliorated completely. On the other hand, multifocal conduction slowing without conduction block preferentially at distal nerve segments and compression‐susceptible sites remained (Table 1), which implied the presence of subclinical and persistent peripheral nerve dysfunction before the onset of the neurological symptoms. As his clinical and NCS findings indicated muscle training‐induced bilateral brachial plexopathy in HNPP, genetic analysis for this disorder was performed, which revealed deletion of the *PMP22* gene. The patient was advised to avoid the causative training, and his neurological symptoms resolved completely within 1 month. He restarted playing baseball and has continued school baseball club activity without recurrence for 7 months.

**Figure 1:**
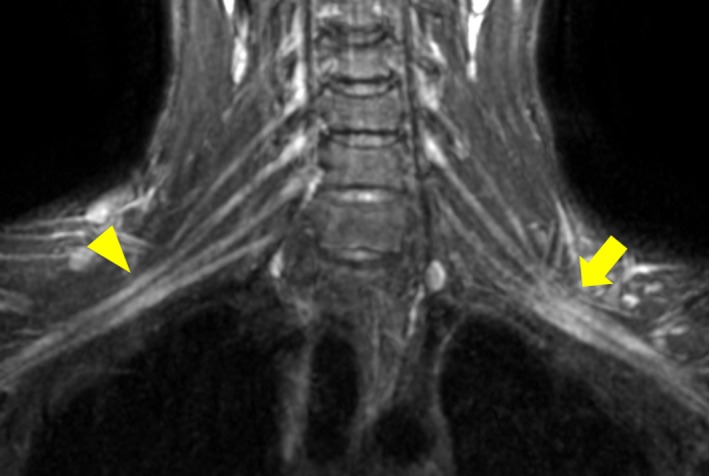
Magnetic resonance imaging of the brachial plexus. Maximum intensity projection imaging of short TI inversion recovery (STIR) (inversion time/repetition time/echo time = 160/2,838/69 ms) indicated enlargement and hyperintensity of bilateral brachial plexuses, predominantly on the left side (arrow: left side, arrowhead: right side)

**Table 1:** Results of nerve conduction studies

	1st NCS (L/R)	2nd NCS (L/R)	Normal values
*Motor nerve conduction*			
Distal motor latency (ms)			
Median	**4.8/5.1**	**4.8/5.0**	<4.1
Ulnar	**4.2/3.7**	**4.2/3.5**	<3.1
Peroneal	**6.9/**N.E	N.E**/6.8**	<5.6
Tibial	**5.8**/N.E	N.E/4.7	<5.1
MCV (m/s)			
Median (wrist–elbow)	55/51	53/54	>51
Ulnar (wrist–below elbow)	52/47	52/50	>51
Ulnar (below–above elbow)	**28/29**	**31/36**	>48
Peroneal (ankle–below fibular head)	32/N.E	N.E/38	>39
Peroneal (below–above fibular head)	42/N.E	N.E/43	>40
Tibial (ankle–popliteal)	38/N.E	N.E/41	>39
CMAP amplitude (mV)			
Median	6.3/8.9	9.0/7.5	>5.1
Ulnar	9.4/11.2	8.1/11.0	>5.5
Peroneal	6.0/N.E	N.E/7.1	>0.9
Tibial	10.5/N.E	N.E/9.2	>6.0
F‐wave latency (ms)			
Median	26.7/31.2	30.9/30.3	<28.5
Ulnar	34.2/32.8	35.2/29.9	<27.7
Peroneal	58.8/N.E	N.E/57.6	<54.1
Tibial	58.4/N.E	N.E/53.8	<51.6
F‐wave occurrence (%)			
Median	81/56	90/70	≥70
Ulnar	56/56	80/80	≥70
Peroneal	56/N.E	N.E/50	No data
Tibial	100/N.E	N.E/100	100
*Sensory nerve conduction*			
SCV (m/s)			
Median (2nd finger–wrist)	**41/38**	**52/48**	>52
Ulnar (5th finger–wrist)	**40/46**	**49/**50	>49
Sural (ankle–calf)	43/N.E	N.E/44	>40
SNAP amplitude (μV)			
Median	19.2/14.4	15.8/13.8	>12.0
Ulnar	10.8/12.8	15.8/11.3	>9.0
Sural	11.2/N.E	N.E/15.8	>6.0

Conduction slowing at distal nerve segments and compression‐susceptive sites is indicated in bold. Normal values are determined according to the results of NCSs in 30 healthy volunteers.

CMAP, compound muscle action potential; L, left side; MCV, motor nerve conduction velocity; NCS, nerve conduction study; N.E, not examined; R, right side; SCV, sensory nerve conduction velocity; SNAP, sensory nerve action potential.

HNPP is an autosomal dominant genetic disease; however, 20%–78% of patients constitute sporadic cases with subclinical asymptomatic relatives and *de novo* onset (Infante et al., 2001). Brachial plexopathy is the third most common clinical phenotype in patients with HNPP following peroneal and ulnar mononeuropathies (Mouton et al., 1999); however, bilateral involvement is unusual (Horowitz et al., 2004; Kim, 2014). Patients sometimes show an atypical clinical phenotype, and a diagnosis of HNPP can therefore be challenging, especially in sporadic cases (Horowitz et al., 2004; Infante et al., 2001), as in our patient.

To date, few patients with HNPP that developed brachial plexopathy after physical training have been reported (Horowitz et al., 2004; Kim, 2014; Mäkelä et al., 2006). All these patients were young‐adult soldiers that developed symptoms during military training. For instance, Horowitz et al. (2004) reported a 21‐year‐old women with HNPP that showed bilateral brachial plexopathy while doing push‐ups and carrying heavy backpack. She repeated military physical training for 3 weeks, which induced axonal degeneration and permanent disability. Mäkelä et al. (2006) evaluated the incidence of compression plexopathy of the shoulder region in 152,095 Finish military conscripts and identified four patients with HNPP that developed brachial plexopathy after backpack carriage. Similarly, Kim (2014) searched the medical records of all South Korean military hospitals between 2011 and 2012 and identified 13 cases with HNPP among 189 patients who developed brachial plexopathy. Among 13 patients with HNPP, 10 cases presented with brachial plexopathy after performing push‐ups.

The case presented here is the first case of an adolescent with HNPP that developed neurological symptoms during muscle training in a school baseball club activity. In addition, the patient developed brachial plexopathy after barbell and plank training, neither of which has been previously reported as a trigger of HNPP. In our patient, the barbell would have compressed the brachial plexus directly, which may have induced left side plexopathy similar to backpack carriage (Horowitz et al., 2004; Kim, 2014; Mäkelä et al., 2006). Barbell loading on the patient's arms would also stretch the brachial plexus indirectly, which may also have caused plexopathy. The plank is a popular core muscle training involving maintenance of a position similar to a push‐up. We speculated that the plank triggered right side brachial plexopathy in our patient through a pathomechanism similar to that induced because of push‐ups (i.e., contraction of the muscles around the brachial plexus against the clavicle, which causes direct compression brachial plexopathy (Kim, 2014)). We presume that conduction slowing at the carpal and cubital tunnels did not induce muscle weakness, as there was neither conduction block nor decrease in amplitude of distal compound muscle potentials. However, some of the conduction slowing at the carpal and cubital tunnels was improved between the 1st and 2nd NCSs. This suggests that barbell and/or plank training induced milder demyelination at these compression‐susceptible sites. In addition, compression of the radial nerve at the spiral groove due to persistent triceps contraction might coexist with and induce left side wrist and finger extensor muscle weakness, although we did not evaluate the radial nerve using NCS.

To the best of our knowledge, there has been only one case of HNPP that was evaluated using MR plexography (Chhabra et al., 2016). The case was that of a 35‐year‐old man with HNPP who had abnormal hyperintense and mildly thickened brachial and lumbosacral plexuses in MR plexography. However, the clinical features of the patient have not been described. The abnormal MR plexographic findings in our patient might reflect demyelination, remyelination, and/or edema, although the precise underlying pathomechanisms are not apparent. Further studies using MR plexography, including serial assessment correspondence with clinical and electrophysiological findings, are needed in patients with HNPP.

Our case indicated that muscle training‐induced brachial plexopathy could be an initial symptom and may be underdiagnosed in adolescents with HNPP. Patients with HNPP might need to focus on particular muscle training exercises, such as barbell lifting, planks, and push‐ups, as repetitive causative training could induce irreversible axonal degeneration (Horowitz et al., 2004; Koike et al., 2005). Further investigations, especially on exercise modalities, are required to verify the benefits and risks of exercise in patients with HNPP.

## References

[brb3783-bib-0001] Chhabra, A. , Carrino, J. A. , Farahani, S. J. , Thawait, G. K. , Sumner, C. J. , Wadhwa, V. , … Lloyd, T. E. (2016). Whole‐body MR neurography: Prospective feasibility study in polyneuropathy and Charcot‐Marie‐Tooth disease. Journal of Magnetic Resonance Imaging, 44, 1513–1521. 27126998 10.1002/jmri.25293

[brb3783-bib-0002] Fowler, T. J. , Danta, G. , & Gilliatt, R. W. (1972). Recovery of nerve conduction after a pneumatic tourniquet: Observations on the hind‐limb of the baboon. Journal of Neurology, Neurosurgery and Psychiatry, 35, 638–647. 10.1136/jnnp.35.5.638 PMC494143 4628467

[brb3783-bib-0003] Garber, C. E. , Blissmer, B. , Deschenes, M. R. , Franklin, B. A. , Lamonte, M. J. , Lee, I. M. , … American College of Sports Medicine . (2011). American College of Sports Medicine position stand. Quantity and quality of exercise for developing and maintaining cardiorespiratory, musculoskeletal, and neuromotor fitness in apparently healthy adults: Guidance for prescribing exercise. Medicine and Science in Sports and Exercise, 43, 1334–1359. 21694556 10.1249/MSS.0b013e318213fefb

[brb3783-bib-0004] Horowitz, S. H. , Spollen, L. E. , & Yu, W. (2004). Hereditary neuropathy with liability to pressure palsy: Fulminant development with axonal loss during military training. Journal of Neurology, Neurosurgery and Psychiatry, 75, 1629–1631. 10.1136/jnnp.2003.029314 PMC1738805 15489403

[brb3783-bib-0005] Infante, J. , García, A. , Combarros, O. , Mateo, J. I. , Berciano, J. , Sedano, M. J. , … Palau, F. (2001). Diagnostic strategy for familial and sporadic cases of neuropathy associated with 17p11.2 deletion. Muscle and Nerve, 24, 1149–1155. 11494267 10.1002/mus.1126

[brb3783-bib-0006] Kim, K. E. (2014). Characteristic features of hereditary neuropathy with liability to pressure palsy (HNPP) presenting with brachial plexopathy in soldiers. Journal of the Neurological Sciences, 346, 174–177. 25175852 10.1016/j.jns.2014.08.018

[brb3783-bib-0007] Koike, H. , Hirayama, M. , Yamamoto, M. , Ito, H. , Hattori, N. , Umehara, F. , … Sobue, G. (2005). Age associated axonal features in HNPP with 17p11.2 deletion in Japan. Journal of Neurology, Neurosurgery and Psychiatry, 76, 1109–1114.

[brb3783-bib-0008] Mäkelä, J. P. , Ramstad, R. , Mattila, V. , & Pihlajamäki, H. (2006). Brachial plexus lesions after backpack carriage in young adults. Clinical Orthopaedics and Related Research, 452, 205–209. 16906084 10.1097/01.blo.0000229338.29277.29

[brb3783-bib-0009] Mouton, P. , Tardieu, S. , Gouider, R. , Birouk, N. , Maisonobe, T. , Dubourg, O. , … Richardson, P. M. (1999). Spectrum of clinical and electrophysiologic features in HNPP patients with the 17p11.2 deletion. Neurology, 52, 1440–1446. 10227632 10.1212/wnl.52.7.1440

[brb3783-bib-0010] Pinheiro, D. S. , Manzano, G. M. , & Nóbrega, J. A. (2008). Reproducibility in nerve conduction studies and F‐wave analysis. Clinical Neurophysiology, 119, 2070–2073. 18621579 10.1016/j.clinph.2008.05.006

[brb3783-bib-0011] Sman, A. D. , Hackett, D. , Fiatarone Singh, M. , Fornusek, C. , Menezes, M. P. , & Burns, J. (2015). Systematic review of exercise for Charcot‐Marie‐Tooth disease. Journal of the Peripheral Nervous System: JPNS, 20, 347–362. 26010435 10.1111/jns.12116

