# Muscle training‐induced bilateral brachial plexopathy in an adolescent with sporadic hereditary neuropathy with liability to pressure palsies

**DOI:** 10.1002/brb3.1231

**Published:** 2019-02-18

**Authors:** Minori Kodaira, Satoshi Kodama, Yui Kamijo, Tomoki Kaneko, Yoshiki Sekijima

In Kodaira, Kodama, Kamijo, Kaneko, and Sekijima (2017), an error was publish in Table 1 footnote. The number of healthy subjects for determining the normal values of nerve conduction studies (NCSs) should be “25” instead of “30”, so it reads “…results of NCSs in 25 healthy volunteers.”

## References

[brb31231-bib-0001] Kodaira, M. , Kodama, S. , Kamijo, Y. , Kaneko, T. , & Sekijima, Y. (2017). Muscle training‐induced bilateral brachial plexopathy in an adolescent with sporadic hereditary neuropathy with liability to pressure palsies. Brain and Behavior, 7(9), e00783 10.1002/brb3.783 28948078 PMC5607547

